# Human Pathogenic Bacteria Detected in Rainwater: Risk Assessment and Correlation to Microbial Source Tracking Markers and Traditional Indicators

**DOI:** 10.3389/fmicb.2021.659784

**Published:** 2021-05-07

**Authors:** Julia K. Denissen, Brandon Reyneke, Monique Waso, Sehaam Khan, Wesaal Khan

**Affiliations:** ^1^Department of Microbiology, Faculty of Science, Stellenbosch University, Stellenbosch, South Africa; ^2^Faculty of Health Sciences, University of Johannesburg, Doornfontein, South Africa

**Keywords:** microbial source tracking markers, human pathogenic bacteria, traditional indicator organisms, QMRA, rainwater

## Abstract

Roof-harvested rainwater (RHRW) was investigated for the presence of the human pathogenic bacteria *Mycobacterium tuberculosis* (*M. tuberculosis*), *Yersinia* spp. and *Listeria monocytogenes* (*L. monocytogenes*). While *Yersinia* spp. were detected in 92% (*n* = 25) of the RHRW samples, and *L. monocytogenes* and *M. tuberculosis* were detected in 100% (*n* = 25) of the samples, a significantly higher mean concentration (1.4 × 10^3^ cells/100 mL) was recorded for *L. monocytogenes* over the sampling period. As the identification of appropriate water quality indicators is crucial to ensure access to safe water sources, correlation of the pathogens to traditional indicator organisms [*Escherichia coli* (*E. coli*) and *Enterococcus* spp.] and microbial source tracking (MST) markers (*Bacteroides* HF183, adenovirus and Lachnospiraceae) was conducted. A significant positive correlation was then recorded for *E. coli* versus *L. monocytogenes* (*r* = 0.6738; *p* = 0.000), and *Enterococcus* spp. versus the *Bacteroides* HF183 marker (*r* = 0.4071; *p* = 0.043), while a significant negative correlation was observed for *M. tuberculosis* versus the *Bacteroides* HF183 marker (*r* = −0.4558; *p* = 0.022). Quantitative microbial risk assessment indicated that the mean annual risk of infection posed by *L. monocytogenes* in the RHRW samples exceeded the annual infection risk benchmark limit (1 × 10^–4^ infections per person per year) for intentional drinking (∼10^–4^). In comparison, the mean annual risk of infection posed by *E. coli* was exceeded for intentional drinking (∼10^–1^), accidental consumption (∼10^–3^) and cleaning of the home (∼10^–3^). However, while the risk posed by *M. tuberculosis* for the two relevant exposure scenarios [garden hosing (∼10^–5^) and washing laundry by hand (∼10^–5^)] was below the benchmark limit, the risk posed by adenovirus for garden hosing (∼10^–3^) and washing laundry by hand (∼10^–3^) exceeded the benchmark limit. Thus, while the correlation analysis confirms that traditional indicators and MST markers should be used in combination to accurately monitor the pathogen-associated risk linked to the utilisation of RHRW, the integration of QMRA offers a more site-specific approach to monitor and estimate the human health risks associated with the use of RHRW.

## Introduction

A conservative estimate predicts that 4% of deaths worldwide and 5.7% of the global burden of disease in disability-adjusted life years (DALYs) could be attributed to water, sanitation and hygiene (WASH) related infectious diseases (Yang et al., 2012). The primary aim of Sustainable Development Goal 6 (SDG6) is thus to ensure that the world population has access to safe and affordable water and adequate sanitation services by 2030 (United Nations, 2018). In an effort to achieve this aim, roof-harvested rainwater (RHRW) is being investigated and applied as an alternative, supplementary water source in many countries around the world, including South Africa (Ahmed et al., 2008b; De Kwaadsteniet et al., 2013). However, while RHRW may be used to augment current water supplies (Gould and Nissen-Petersen, 1999), rainwater is exposed to various contamination sources as it traverses the air (e.g., bioaerosols) and during the harvesting process (e.g., debris and animal faecal matter on the catchment surface) (Hamilton et al., 2019).

It is thus well documented that the microbiological quality of RHRW is sub-standard and numerous research groups have reported on the detection of traditional indicator organisms (using culture-based analysis) such as total coliforms, faecal coliforms, *Escherichia coli* (*E. coli*) and enterococci species in rainwater (Kaushik et al., 2014; Waso et al., 2018; Hamilton et al., 2019). While the analysis of the indicator groups is routine in water quality monitoring, amongst other pitfalls, a poor correlation has been recorded between traditional indicator organisms and potentially pathogenic microorganisms (Harwood et al., 2005, 2014; Field and Samadpour, 2007). Researchers are thus investigating the use of microbial source tracking (MST) markers to monitor and detect faecal contamination within environmental water samples (Ahmed et al., 2016). Microbial source tracking methods have greatly improved the capacity to detect microorganisms that are host-specific to animals, that occur in water and sediments and unlike faecal indicator bacteria, MST markers are able to differentiate between several sources of faecal contamination (Bradshaw et al., 2016). A few of the common MST markers include the *Enterococcus esp* gene, enterovirus, *Bifidobacterium* spp., human-specific *Bacteroides* HF183, human adenovirus and polyomavirus. Ideal characteristics of these MST markers include: specificity to the target host-group; the marker should be geographically and temporally stable in the target host-group, and; the decay rates of the markers and pathogens present in the relevant water sources should correlate (Ahmed et al., 2015). Moreover, advances in water quality monitoring techniques such as molecular viability and whole community analysis, renders culture-based analyses superfluous and ensures the accurate detection and quantification of MST markers and waterborne pathogens. Using quantitative PCR (qPCR), Savichtcheva et al. (2007) observed a significant positive correlation between the human *Bacteroides* MST marker versus *Salmonella*, whilst Viau and Boehm (2011) observed a significant correlation between the human *Bacteroides* MST marker and *Leptospira*.

Using molecular detection methods, numerous studies have also identified a variety of opportunistic and pathogenic microorganisms in RHRW. These frequently detected microorganisms include *Legionella pneumophila*, *Pseudomonas aeruginosa*, *Klebsiella pneumoniae* and *Salmonella* spp. (Hamilton et al., 2019). Ahmed et al. (2017); Strauss et al. (2019) and Reyneke et al. (2020) then employed Illumina amplicon-based sequencing and ethidium monoazide bromide (EMA)-Illumina analysis, to investigate the whole bacterial community in RHRW. Correspondingly, many of the frequently detected bacterial genera such as *Legionella*, *Pseudomonas* etc. formed part of this indigenous or core microbial rainwater group. However, a high frequency of detection percentage was also obtained for genera such as *Mycobacterium*. This genus includes pathogens known to cause serious disease in humans, which is concerning as the utilisation of RHRW as an alternative water source may pose a health risk to the end-user communities. *Mycobacterium tuberculosis* (*M. tuberculosis*) was thus predominantly focused on in the current study and as *Yersinia* spp. and *Listeria monocytogenes* (*L. monocytogenes*) have previously been detected in rainwater, these bacterial pathogens were also included (Dobrowsky et al., 2014; Jongman and Korsten, 2016).

The Gram-negative coccobacillus *Yersinia* genus forms part of the *Yersiniaceae* family and is comprised of 11 species, three of which are pathogenic to humans: *Y. pseudotuberculosis*, *Y. enterocolitica* and *Y. pestis*. *Y. pestis* is responsible for causing three different forms of the highly infectious disease known as the plague (pneumonic, bubonic and septicaemic) (Pechous et al., 2016). The plague is transmitted by fleas to various hosts by blood feeding, or by regurgitative transmission of the bacteria (once it has grown in the form of a cohesive biofilm) from the flea host into the bite of the receiving organism and can persist for extended periods of time at low levels in enzootic cycles (Eisen et al., 2006; Hinnebusch et al., 2017; Bosio et al., 2020). Although limited research is available on the presence of *Yersinia* spp. in RHRW, Dobrowsky et al. (2014) identified the *Yersinia* genus in rainwater using conventional PCR analysis. There is thus a high probability that these species are present in RHRW systems as bird and rodent faecal matter are often detected on roofing systems and might wash into the harvesting tanks during a rain event. Similarly, *M. tuberculosis*, which causes tuberculosis, can survive and adapt to hostile or extreme environmental conditions (Cook et al., 2009). It is also capable of adapting to a variety of intracellular human systems such as dendritic cells and macrophages (Cook et al., 2009). However, despite its poor geographical characterisation in terms of abundance in various environments (King et al., 2017), as previously indicated, using Illumina and EMA-Illumina whole-community analysis, the *Mycobacterium* genus was identified as one of the primary frequently detected genera in rainwater (Ahmed et al., 2017; Strauss et al., 2019; Reyneke et al., 2020). This is a matter of serious concern as 360 000 people were diagnosed with tuberculosis in South Africa in 2019 [World Health Organisation (WHO), 2019] and 781 tuberculosis cases are reported amongst every 100 000 individuals each year (Tadokera et al., 2020). *L. monocytogenes* is a Gram-positive, psychotropic bacteria that has been detected in the environment and typically occurs in most raw foods. It is responsible for causing listeriosis and from 2017 to 2018, *L. monocytogenes* sequence type 6 was associated with a listeriosis outbreak in South Africa, that was described by the WHO as the biggest outbreak ever recorded worldwide (Smith et al., 2019). Jongman and Korsten. (2016) then detected *L. monocytogenes* (using selective culture-based analysis combined with matrix-assisted laser desorption/ionisation time of flight mass spectrometry) in RHRW samples collected from three rural South African villages which rely on RHRW as an alternative water source.

Roof-harvested rainwater thus has the potential to expose vulnerable end-user communities to a myriad of microbial pathogens and opportunistic pathogens (Low, 2002). Quantitative microbial risk assessment (QMRA) can therefore be implemented as a health risk assessment tool and is a technique that has been used for more than two decades to estimate the pathogen-associated risk in drinking water (Pecson et al., 2017). This technique is comprised of four stages, namely (i) hazard identification, (ii) exposure assessment, (iii) dose-response modelling and (iv) risk characterisation. The combination of these stages allows for the construction of a prediction-based analysis model that elucidates the potential health risk associated with specific pathogens based on exposure scenarios associated with a particular environment or activity (Owens et al., 2020). Moreover, using a QMRA framework, it is envisaged that the gap between the level of pathogens in a water source and the treatment required to effectively reduce pathogen-associated risk, can be bridged (Sano et al., 2019).

The primary aim of this study was thus to explore a consortium of RHRW tanks located in a sustainable housing project in Kleinmond, South Africa for the human pathogenic bacterial species *M. tuberculosis*, *Yersinia* spp. and *L. monocytogenes*. Additionally, the presence of the human bacterial pathogens (*M. tuberculosis*, *Yersinia* spp. and *L. monocytogenes*) was correlated to the presence of traditional indicator organisms (i.e., *E. coli* and *Enterococcus* spp.) and MST markers (i.e., *Bacteroides* HF183, adenovirus and Lachnospiraceae). The *Bacteroides* HF183 marker was selected as the HF183 primer set occurs in all *Bacteroides* strains of human origin. This marker thus has high specificity for the detection of human faecal matter and sewage in environmental waters (Harwood et al., 2014). Similarly, both Lachnospiraceae and adenovirus have been investigated as indicators of sewage and faecal pollution in environmental waters and could therefore provide valuable information on the health risks associated with the use of various water sources (Newton et al., 2011; McLellan et al., 2013; Sidhu et al., 2013; Rusiñol et al., 2016; Waso et al., 2018). Quantitative PCR analyses were used to identify and quantify the target bacterial pathogens, MST markers and indicator groups, whereafter the health risk associated with the utilisation of the RHRW for potable and various domestic activities (e.g., cleaning the house, laundry, etc.), in the target community, was determined.

## Materials and Methods

### Sampling Site

The Kleinmond Housing Scheme, Western Cape, South Africa (GPS co-ordinates: 34°20.11′ 81″ S; 19° 00.59′ 74″ E), was used as the sampling site. The sustainable housing project was conceptualised in 2007 in a collaboration between the Overstrand Local Municipality, the Council for Scientific and Industrial Research, the Department of Science and Technology and the Western Cape Provincial Department of Human Settlements. Within this community are 411 houses (40 m^2^ each), each fitted with a 2000 L aboveground polyethylene rainwater harvesting tank. A random sampling technique was implemented to select five houses, designated A to E (with functioning rainwater harvesting tanks installed), from a collection of houses used in the Dobrowksy et al. (2014) study. Sampling was conducted once a week for five consecutive weeks (August to September 2020), with 5 L of rainwater collected from each RHRW tank (*n* = 25) using sterile polypropylene bottles as previously described by Waso et al. (2018).

The temperature of each sample was measured on-site using a hand-held mercury thermometer; the pH, total dissolved solids, and electrical conductivity were determined using a hand-held Milwaukee Instruments MI806 meter, and the dissolved oxygen was measured using a hand-held Milwaukee Instruments M600 meter (Spraytech, South Africa). All physico-chemical parameters are outlined in Supplementary Table 1 (Supplementary Material). The daily ambient temperature and rainfall data for the duration of the sampling period was obtained from the South African Weather Services (Pretoria, South Africa). A visual representation of the daily ambient temperature and rainfall data is shown in Supplementary Figure 1 (Supplementary Material).

### Rainwater Concentration, EMA Treatment and DNA Extraction for the Detection of Target Pathogens and Indicator Organisms

One litre of each RHRW sample (*n* = 25) was subjected to flocculation as previously described by Dobrowsky et al. (2015). The flocculated samples were filtered through non-charged mixed ester membrane filters with a pore size of 0.45 μM (Merck, Millipore, Billerica, MA, United States) and each filter was placed in a 9 cm petri dish containing 1.5 mL citrate buffer (0.3 M, pH 3.5) (Saarchem, Durban, South Africa) and gently agitated using an orbital platform shaker to remove the cells from the filter. Thereafter, each 1.5 mL concentrated sample was centrifuged at 16,000 × *g* for 5 min and the resulting pellet was subjected to 6 μM EMA (Biotium, Hayward, CA, United States) treatment as previously described by Reyneke et al. (2017). Ethidium monoazide bromide intercalates with the DNA of cells with compromised membranes or with extracellular DNA. Upon photoactivation, EMA covalently binds to the DNA, which inhibits their amplification in quantification assays (e.g., qPCR) (Emerson et al., 2017). Treatment with EMA was thus done so that the detected gene copies for the pathogens and indicator organisms could be converted into viable cells (may be detected using culture-based analysis) for use in the QMRA analysis. Following EMA treatment, the supernatant containing residual EMA was removed and total genomic DNA extractions (i.e., DNA from intact and presumed viable cells) were performed on the remaining pellet for each of the RHRW samples (*n* = 25) using the *Quick*-DNA^TM^ Fecal/Soil Microbe Miniprep Kit (Zymo Research, Irvine, CA, United States) according to the manufacturer’s instructions.

### Rainwater Concentration and DNA Extraction for the Detection of MST Markers

Preliminary comparative analysis of EMA-qPCR (intact and presumed viable cells) versus qPCR (whole or total DNA, without EMA), indicated that the EMA treatment may have influenced the detection of the MST markers in the RHRW. Therefore, conventional qPCR was used for the detection of the MST markers (i.e., *Bacteroides* HF183, Lachnospiraceae, adenovirus) within the RHRW samples. One litre of each RHRW sample (*n* = 25) was concentrated as described in Section “Rainwater Concentration, EMA Treatment and DNA Extraction for the Detection of Target Pathogens and Indicator Organisms.” Thereafter, each 1.5 mL concentrated sample was centrifuged at 16,000 × *g* for 5 min and total genomic DNA extractions were performed on the remaining pellet for each of the RHRW samples (*n* = 25) using the *Quick*-DNA Fecal/Soil Microbe Miniprep Kit (Zymo Research) according to the manufacturer’s instructions.

### Quantitative PCR Analyses

All qPCR analyses were conducted using a LightCycler^®^96 Instrument (Roche Diagnostics, Mannheim, Germany) and the FastStart Essential DNA Green Master/FastStart Essential DNA Probes Master (Roche Diagnostics) in order to quantify gene copies of the target pathogens *M. tuberculosis*, *Yersinia* spp. and *L. monocytogenes*, as well as the MST markers *Bacteroides* HF183, adenovirus and Lachnospiraceae, and the indicator organisms *E. coli* and *Enterococcus* spp. in the RHRW samples. The primers and cycling parameters for each target organism are outlined in Table 1. Each qPCR assay was performed in duplicate. The reaction mixture (final volume of 20 μL) for all the qPCR assays, except the Lachnospiraceae assay, consisted of 10 μL FastStart Essential DNA Green Master (1X), 0.4 μL of the forward and reverse primers (0.2 μM) and 5 μL template DNA. For the Lachnospiraceae assay, the reaction mixture consisted of 10 μL (1X) FastStart Essential DNA Probes Master, 2 μL primer-probe mixture [1 μM of each primer and 0.08 μM of the probe], 3 μL PCR-grade water and 5 μL template DNA. All DNA samples were diluted 10-fold before analysis with the respective qPCR assays in order to minimise PCR inhibitors (Reyneke et al., 2017). For each qPCR reaction, a negative control of sterile milliQ was included in the analysis, while melt curve analysis was included for all SYBR^®^ Green qPCR assays to verify the specificity of the primer sets (temperature increase from 65 to 97 °C at 0.2°C/s and continuous fluorescent signal acquisition at 5 readings/°C) (Reyneke et al., 2017).

**TABLE 1:** Conventional PCR and qPCR primers, cycling parameters and PCR product size of the organisms screened for in the RHRW samples.

Organism	Primers	Primer sequence (5′-3′)	[Primer] for conventional PCR	Conventional PCR cycling parameters	qPCR cycling parameters	Target gene (bp)	Mean copies per cell	References
*Mycobacterium tuberculosis*	MTP t8-F MTP t9-R	GTGCGGATGGTCGCAGAGAT CTCGATGCCCTCACGGTTCA	0.2 μM	3 min at 95°C; 40 cycles of 94°C for 1.5 min, 65°C for 2 min, 72°C for 3 min	3 min at 95°C; 40 cycles of 94°C for 1.5 min, 65°C for 2 min, 72°C for 3 min	*orfA* (541)	15^a^	Kox et al., 1994
*Yersinia* spp.	227Fmod 669R	GTCTGGGCTTTGCTGGTC GCGTCGTATTTAGCACCAACG	0.8 μM	5 min at 95°C; 40 cycles of 94°C for 20 s, 60°C for 20 s, 72°C for 15 s	5 min at 95°C; 40 cycles of 94°C for 20 s, 60°C for 20 s, 72°C for 15 s	*ompF* (465)	1^b^	Stenkova et al., 2008
*Listeria monocytogenes*	LIS-F LIS-R	TCATCGACGGCAACCTCGG TGAGCAACGTATCCTCCAGAGT	0.3 μM	7 min at 95°C; 40 cycles of 50 s at 95°C, 40 s at 54°C, 50 s at 72°C, 5 min at 72°C	7 min at 95°C; 40 cycles of 95°C for 50 s, 54°C for 40 s, 72°C for 50 s	*prfA* (217)	2^b^	Germini et al., 2009
*Bacteroides* HF183	HF183F HF183R	ATCATGAGTTCACATGTCCG TACCCCGCCTACTATCTAATG	0.25 μM	95°C for 4 min; 40 cycles of 95°C for 30 s, 53°C for 1 min, 72°C for 2 min; final elongation at 72°C for 10 min	95°C for 10 min; 40 cycles of 95°C for 30 s, 53°C for 1 min, 60°C for 1 min	16 S rRNA (86)	7^c^	Seurinck et al., 2005
*E. coli*	784F 866R	GTGTGATATCTACCCGCTTCGC AGAACGGTTTGTGGTTAATCAGGA	0.5 μM	95°C for 10 min; 50 cycles of 95°C for 15 s, 60°C for 1 min, final elongation at 72°C for 10 min	95°C for 10 min; 50 cycles of 95°C for 15 s, 60°C for 1 min	*uidA* (80)	1^d^	Frahm and Obst, 2003
*Enterococcus* spp.	ECST784F ENC854R	AGAAATTCCAAACGAACTTG CAGTGCTCTACCTCCATCATT	0.5 μM	95°C for 10 min; 50 cycles of 95°C for 15 s, 60°C for 1 min, final elongation at 72°C for 10 min	95°C for 10 min; 50 cycles of 95°C for 15 s, 60°C for 1 min	23 S rRNA (80)	6^c^	Frahm and Obst, 2003
Adenovirus	AQ1 AQ2	GCCACGGTGGGGTTTCTAAACTT GCCCCAGTGGTCTTACATGCACATC	0.3 μM	94 °C for 2 min; 35 cycles of 94 °C for 30 s, 55 °C for 1 min, 72 °C for 1 min; final elongation at 72 °C for 7 min	95 °C for 10 min; 55 cycles of 95 °C for 3 s, 55 °C for 10 s, 65 °C for 1 min	Hexon (110)	1^b^	Heim et al., 2003
Lachnospiraceae	Lachno2 FWD Lachno2 REV Lachno2 probe	TTCGCAAGAATGAAACTCAAAG AAGGAAAGATCCGGTTAAGGATC 6-carboxyfluoroscein (6-FAM)-ACCAAGTCTTGACATCCG – minor groove binder (MGB)	200 μM	95 °C for 10 min; 40 cycles of 95 °C for 15 s, 60 °C for 1 min, 72 °C for 1 min; final elongation at 72 °C for 10 min	50 °C for 2 min, 95 °C for 10 min; 55 cycles of 95 °C for 15 s, 60 °C for 1 min	16S rRNA (144)	5^c^	Newton et al., 2011

*^a^Wall et al. (1999). ^b^National Center for Biotechnology Information (NCBI) (2020). ^c^Stoddard et al. (2015). ^d^Reyneke et al. (2020).*

Standard curves for the qPCR assays were generated using the methodology outlined in Reyneke et al. (2017). Briefly, conventional PCR assays (Table 1) were performed to amplify the respective target genes using positive control DNA [*E. coli* ATCC 13706, *Enterococcus faecalis* (clinical isolate), lyophilised adenovirus (Coris Bioconcept, Gembloux, Belgium), *L. monocytogenes* ATCC 13932 and *Yersinia enterocolitica* subsp. *enterocolitica* ATCC 27729] or DNA extracted from an influent sewage sample collected from a local wastewater treatment plant (*M. tuberculosis*, Lachnospiraceae and *Bacteroides* HF183) (Supplementary Table 2, Supplementary Material). A negative control of sterile milliQ was utilised for each PCR reaction.

A standard curve was generated by preparing serial 10-fold dilutions (10^9^ to 10^0^ gene copies/μL) of the PCR products. The lower limit of detection (LLOD) for each qPCR assay was reported as the lowest number of gene copies that was consistently detected in the standard curve (Dobrowsky et al., 2016). The Roche LightCycler^®^96 Software version 1.1 was utilised for the analysis of the qPCR performance characteristics of the assays.

### Quantitative Microbial Risk Assessment

#### Hazard Identification and Quantification of Target Pathogens

Based on the detection frequency of the target pathogens, indicators and MST markers obtained using qPCR analysis (Section “Quantitative PCR Analyses”) and the availability of applicable dose-response models, *E. coli* (representative traditional indicator organism), adenovirus (representative MST marker), *L. monocytogenes* and *M. tuberculosis* were selected as the target organisms for the health risk assessment of the RHRW. For use in the QMRA analyses, the detected gene copies (Section “Quantitative PCR Analyses”) were converted to gene copies/100 mL of the original RHRW sample as outlined by Waso et al. (2018). The gene copies/100 mL were then converted to cell equivalents (cells/100 mL) by utilising the number of copies of the target gene present within the host (Table 1). All final concentrations for the EMA-qPCR (intact and presumed viable cells) and conventional qPCR (whole or total DNA) analyses are thus presented as equivalent cells/100 mL original RHRW sample.

#### Exposure Assessment

The major exposure routes associated with the use of RHRW for several domestic activities in the Kleinmond Housing Scheme site were identified by consulting social survey data reported by Dobrowksy et al. (2014). These activities include washing laundry by hand, cleaning of the home, garden hosing, garden work, washing/bathing, intentional drinking and accidental consumption. The various exposure scenarios (which includes the exposure volume and frequency of occurrence) that were evaluated in the present study are outlined in Supplementary Table 3 (Supplementary Material).

The formulae used for the calculation of ingestion/inhalation dose, as well as descriptions of the various exposure routes are outlined in Reyneke et al. (2020). The concentration of each respective target organism was obtained from the results of the qPCR analyses. Based on the detection frequency of pathogenic *E. coli* in RHRW from previous studies, the fraction of *E. coli* presumed to be human infectious was set at 0.005–0.1 (Reyneke et al., 2020). Additionally, the fraction of detected adenovirus, *L. monocytogenes* and *M. tuberculosis* assumed to be infectious to humans were 5.88 × 10^–4^, 1.00 and 0.66–1.00, respectively (Buchanan et al., 1997; World Health Organisation, 2004; Lyautey et al., 2007; Schijven et al., 2019).

#### Dose Response

Dose-response models are a set of mathematical expressions which illustrate the probability that an individual will experience an adverse health effect (e.g., infection, death) following exposure to an infectious organism. These models are specifically fitted to the adverse health effects observed in animals or humans that have been exposed to varying doses of infectious microorganisms (Haas et al., 1999; Jones and Su, 2015). Two of the most commonly used dose-response models are the exponential and beta-poisson models. The exponential model assumes that the probability of an organism causing infection is independent of organism dosage, whereas the beta-poisson model assumes that infectivity of an organism is dependent on dose (Buchanan et al., 2000). The exponential dose-response model (Eq. 1) was used to calculate the risk of infection linked to the presence of pathogenic adenovirus (inhalation exposure), *M. tuberculosis* (inhalation exposure) and *L. monocytogenes* (ingestion exposure) within the RHRW samples for various exposure scenarios (Couch et al., 1966; Buchanan et al., 1997; Jones et al., 2009):

(1) $$P_{\inf}=1-\exp^{\left(-kd\right)}$$

where *P*_*inf*_ is the probability of infection following a single exposure, *k* is the parameter which describes the probability of a pathogen surviving the host defence to initiate infection and *d* is the dose of microorganisms (number of microorganisms that is inhaled/ingested).

The beta-Poisson dose-response model (Eq. 2) was then employed to calculate the risk of infection linked to the presence of enteroinvasive *E. coli* (ingestion exposure) within the RHRW samples for various exposure scenarios (Haas et al., 1999; Ryan et al., 2014):

(2) $$P_{inf}=1-{\left(1+\frac{d}{N50}\left(2^{\frac{1}{\alpha }}-1\right)\right)}^{-\alpha }$$

where *P*_*inf*_ is the probability of getting infected following a single exposure event, *d* is the dosage of microorganisms (number of microorganisms that is ingested), *α* is a shape factor and *N*_50_ is the median infective dosage. All parameters associated with the dose-response models are outlined in Table 2.

**TABLE 2:** Monte Carlo simulation dose response input parameters for the target pathogens.

Organism	Variables and distribution*	Dose response model (DRM)	DRM Background	References
Adenovirus	B_C_: β = 0.351; η = 41.458 (Weibull) *IF*_%_: 5.88 × 10^–4^ (Uniform)	Exponential k = 6.07 × 10^–1^	M: Human Ex: Inhalation R: Infection	Couch et al., 1966
*E. coli* (Enteroinvasive *E. coli*)	B_C_: μ = 3.102; σ = 1.070 (Normal) *IF*_%_: 0.005 to 0.10 (Uniform)	Beta-Poisson N_50_ = 2.11 × 10^6^ α = 1.55 × 10^–1^	M: Human Ex: Ingestion R: Infection with positive stool isolation	Haas et al., 1999; Reyneke et al., 2020; Ryan et al., 2014
*L. monocytogenes*	B_C_: μ = 2.253; σ = 0.902 (Lognormal) *IF*_%_: 1.00 (Point)	Exponential k = 1.18 × 10^–10^	M: Human Ex: Ingestion R: Infection	Buchanan et al., 1997; Lyautey et al., 2007
*M. tuberculosis*	B_C_: μ = −3.110; σ = 0.652 (Lognormal) *IF*_%_: 0.66 to 1.00 (Uniform)	Exponential k = 2.18 × 10^–2^	M: Human Ex: Inhalation R: Infection	Jones et al., 2009

**FIGURE 1:**
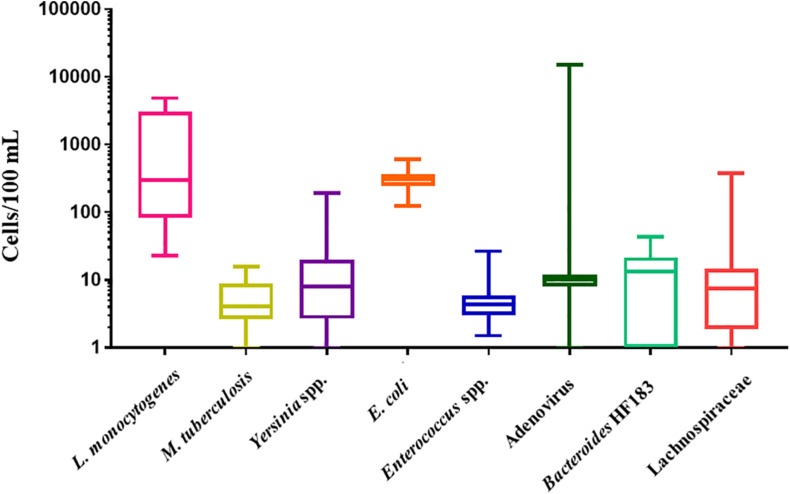
Box and whiskers plot of the concentration (cells/100 mL) for *L. monocytogenes*, *M. tuberculosis*, *Yersinia* spp., *E. coli*, *Enterococcus* spp., adenovirus, *Bacteroides* HF183, and Lachnospiraceae. The whiskers illustrate the minimum and maximum, the outer box illustrates the 1st and 3rd quartiles, and the inner line illustrates the median.

B_c_, bacterial concentration; IF_%_, human infectious fraction of target organism; M, model; Ex, exposure; R, response. *For the QMRA analysis, the Minitab^®^ 19 statistical software package was used to calculate the distribution of each target pathogens’ concentration based on the EMA-qPCR and conventional qPCR data (Figure 1).

#### Risk Characterisation

Lastly, risk characterisation was conducted, whereby the likelihood of infection was calculated for each target pathogen and the corresponding exposure routes as described in Table 2. This was expressed as likely numbers of infections per 10 000 persons per year as previously described by Haas et al. (1999), using Eq. 3:

(3) $$P=1-{\left(1-P_{\text{inf}}\right)}^{n}$$

where *P* is the probability of infection following *n* exposure events per year, based on the previously calculated exposure probability of infection (*P*_*inf*_).

Each exposure scenario was simulated using Monte Carlo analysis in RStudio (version 1.0.153) using 500 000 iterations. Throughout the analyses, the different dose parameters [e.g., pathogen concentrations (Table 2) and ingestion volumes (Supplementary Table 3), amongst others] and exposure events per year (Supplementary Table 3) were sampled randomly based on the corresponding distribution of each parameter. However, the annual risk of infection for adenovirus and *M. tuberculosis* were only determined for two exposure scenarios: garden hosing (aerosol inhalation) and washing laundry by hand (aerosol ingestion), as inhalation of these organisms is the primary route of infection (World Health Organisation, 2005; Fennelly and Jones-López, 2015).

### Statistical Analyses

The relationships between the detected bacterial pathogens, indicator organisms and MST markers enumerated using qPCR analysis, were investigated using Pearson’s correlation analysis and further investigated using Cluster analysis with Ward’s method in Statistica^TM^ version 12.5 (2014). The Cluster analysis with Ward’s method was specifically applied to visualise the relatedness of the detected bacterial pathogens, indicator organisms and MST markers in the RHRW samples (Waso et al., 2018). Cluster analysis is used to illustrate correlations between organisms. A stronger positive correlation (i.e., a higher correlation coefficient between two organisms), will be represented by a lower linkage distance on a dendrogram (Tilevik, 2017).

## Results

### Molecular Viability Quantification (EMA-qPCR) of the Target Pathogens and Indicator Organisms in the RHRW Samples

The quantification of intact *L. monocytogenes*, *M. tuberculosis* and *Yersinia* spp. cells as well as the indicator organisms *E. coli* and *Enterococcus* spp. in the RHRW samples was investigated using EMA-qPCR (intact and presumed viable cells) analysis. The respective performance characteristics of the EMA-qPCR analyses are outlined in Supplementary Table 4 (Supplementary Material).

The LLOD for *L. monocytogenes* (*prfA* gene) was 6 gene copies/μL. *L. monocytogenes* was detected in all RHRW samples (100%, *n* = 25) at a mean concentration of 1.4 × 10^3^ cells/100 mL (Figure 1). The lowest concentration of *L. monocytogenes* was detected in sampling 2 with the cell counts in the RHRW tanks ranging from 23 cells/100 mL to 1.8 × 10^3^ cells/100 mL, while the highest concentration of *L. monocytogenes* was detected in sampling 3, with the cell counts ranging from 2.5 × 10^3^ cells/100 mL to 4.9 × 10^3^ cells/100 mL. The LLOD for *M. tuberculosis* (*orfA* gene) was 3 gene copies/μL. *M. tuberculosis* was detected in all RHRW samples (100%, *n* = 25) at a mean concentration of 6 cells/100 mL (Figure 1). The lowest concentration of *M. tuberculosis* was detected in sampling 5, with the cell counts in the RHRW tanks ranging from 1 cell/100 mL to 4 cells/100 mL, while the highest concentration of *M. tuberculosis* was detected in sampling 2 with the cell counts ranging from 4 cells/100 mL to 16 cells/100 mL. The LLOD for *Yersinia* spp. (*ompF* gene) was 11 gene copies/μL. *Yersinia* spp. were detected in 23 of the RHRW samples (92%, *n* = 25) at a mean concentration of 24 cells/100 mL (Figure 1). The lowest concentration of *Yersinia* spp. cells was detected in sampling 5 with the cell counts in the RHRW tanks ranging from 2 cells/100 mL to 11 cells/100 mL, while the highest concentration of *Yersinia* spp. was detected in sampling 3 with the cell counts ranging from 10 cells/100 mL to 1.5 × 10^2^ cells/100 mL. The LLOD for *E. coli* (*uid*A gene) was 2 gene copies/μL. *E. coli* was detected in all RHRW samples (100%, *n* = 25) at a mean concentration of 3.1 × 10^2^ cells/100 mL (Figure 1). The lowest concentration of *E. coli* was detected in sampling 2 with the cell counts in the RHRW tanks ranging from 1.4 × 10^2^ cells/100 mL to 2.9 × 10^2^ cells/100 mL, while the highest concentration of *E. coli* was detected in sampling 3 and ranged from 3.3 × 10^2^ cells/100 mL to 6.1 × 10^2^ cells/100 mL. The LLOD for *Enterococcus* spp. (23S rRNA gene) was 1 gene copy/μL. *Enterococcus* spp. were detected in all RHRW samples (100%, *n* = 25) at a mean concentration of 6 cells/100 mL (Figure 1). The lowest concentration of *Enterococcus* spp. was detected in sampling 2 with the cell counts in the RHRW tanks ranging from 2 cells/100 mL to 5 cells/100 mL, while the highest concentration of *Enterococcus* spp. was detected in sampling 5 and ranged from 2 cells/100 mL to 27 cells/100 mL.

### Molecular Quantification (qPCR) of the MST Markers in the RHRW Samples

The performance characteristics of the qPCR (whole or total DNA) analyses of the MST markers adenovirus, Lachnospiraceae and *Bacteroides* HF183 are outlined in Supplementary Table 4 (Supplementary Material).

The LLOD for *Bacteroides* HF183 (16S rRNA gene) was 3 gene copies/μL. *Bacteroides* HF183 was detected in 19 of the RHRW samples (76%, *n* = 25) at a mean concentration of 13 cells/100 mL (Figure 1). The lowest concentration of *Bacteroides* HF183 was detected in sampling 2 with the cell counts in the RHRW tanks ranging from 0 cells/100 mL to 16 cells/100 mL, while the highest concentration of *Bacteroides* HF183 cells was detected in sampling 5 and ranged from 0 cells/100 mL to 44 cells/100 mL. The LLOD for adenovirus (Hexon gene) was 5 gene copies/μL. Adenovirus was detected in 24 of the RHRW samples (96%, *n* = 25) at a mean concentration of 6.6 × 10^2^ cells/100 mL (Figure 1). The lowest concentration of adenovirus was detected in sampling 1 with the cell counts in the RHRW tanks ranging from 0 cells/100 mL to 9 cells/100 mL, while the highest concentration of adenovirus cells was detected in sampling 3 and ranged from 9 cells/100 mL to 1.5 × 10^4^ cells/100 mL. The LLOD for Lachnospiraceae (16S rRNA gene) was 1 gene copy/μL. Lachnospiraceae was detected in 20 of the RHRW samples (80%, *n* = 25) at a mean concentration of 24 cells/100 mL (Figure 1). The lowest concentration of Lachnospiraceae was detected in sampling 4 with the cell counts in the RHRW tanks ranging from 0 cells/100 mL to 10 cells/100 mL, while the highest concentration of Lachnospiraceae cells was detected in sampling 2 and ranged from 2 cells/100 mL to 3.7 × 10^2^ cells/100 mL.

### Correlation Between the Target Pathogens, MST Markers and Indicator Organisms

**FIGURE 2:**
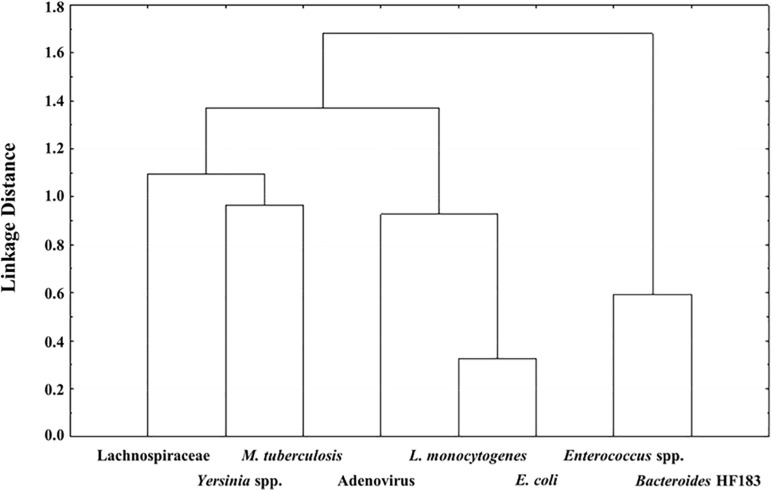
Dendrogram of the Cluster Analysis with Ward’s Methods of target pathogens versus the MST markers and indicator organisms detected in the RHRW samples.

Pearson’s correlation and Cluster analysis was used to correlate and visualise the relatedness of the pathogenic bacterial species, indicator organisms and MST markers detected in the RHRW samples (Table 3 and Figure 2). Results indicated that a significant positive correlation was recorded for *E. coli* versus *L. monocytogenes* (*r* = 0.6738; *p* = 0.000); and *Enterococcus* spp. versus the *Bacteroides* HF183 marker (*r* = 0.4071; *p* = 0.043), while a significant negative correlation was recorded for *M. tuberculosis* versus the *Bacteroides* HF183 marker (*r* = −0.4558; *p* = 0.022) (Table 3). However, despite no significant correlation being observed, based on the cluster analysis, adenovirus was then related to *E. coli* (*r* = 0.1938; *p* = 0.353) and *L. monocytogenes* (*r* = 0.2517; *p* = 0.225) (Figure 2). Additionally, the MST marker Lachnospiraceae clustered with the target pathogens *M. tuberculosis* (*r* = −0.0445; *p* = 0.833) and *Yersinia* spp. (*r* = −0.0810; *p* = 0.700) (Figure 2).

**TABLE 3:**
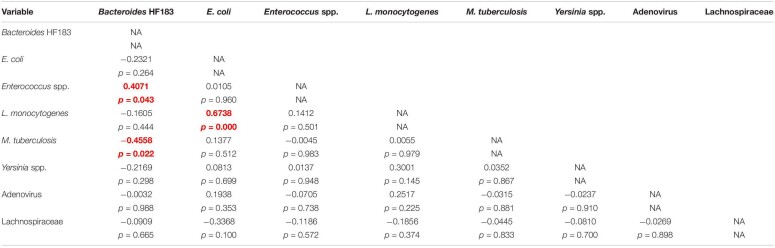
Summary of the correlations observed between the target pathogens, MST markers and indicator organisms detected in the RHRW samples.

*Significant correlations (p < 0.05) are indicated in red and bold.*

### Health Risk Associated With Utilising the RHRW

**FIGURE 3:**
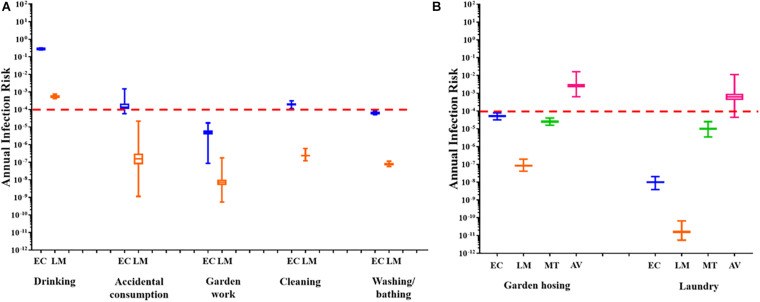
**(A)** Annual health risk associated with the use of RHRW in the community for ingestion scenarios based on the presence of *E. coli* (EC; **blue**) and *L. monocytogenes* (LM; **orange**). **(B)** Annual health risk associated with the use of RHRW in the community for inhalation scenarios based on the presence of *E. coli* (EC; **blue**), *L. monocytogenes* (LM; **orange**), *M. tuberculosis* (MT; **green**) and adenovirus (AV; **pink**). The whiskers illustrate the minimum and maximum, the outer box illustrates the 1st and 3rd quartiles, and the inner line illustrates the median. The benchmark limit (1 × 10^– 4^) is indicated by the dashed red line.

The annual infection risks linked to the utilisation of untreated RHRW for each exposure scenario, based on the presence of pathogenic adenovirus, *L. monocytogenes*, *M. tuberculosis* and *E. coli* (Figure 3) were determined. This was done by comparing all annual risks to a hypothetical benchmark value of 1 × 10^–4^ which represents the benchmark annual risk of infection for drinking water (Regli et al., 1991).

For all the sampling sessions, the mean annual risk of infection posed by *E. coli* for garden hosing (∼10^–4^), garden work (∼10^–5^), washing laundry by hand (10^–8^) and washing/bathing (∼10^–4^) was below the annual infection risk benchmark limit (Figures 3A,B). Similarly, the mean annual risk of infection posed by *L. monocytogenes* for garden hosing (∼10^–7^), garden work (∼10^–8^), washing laundry by hand (10^–11^) and washing/bathing (∼10^–7^) was below the annual infection risk benchmark limit (Figures 3A,B). In comparison, while the annual risk of infection posed by *L. monocytogenes* for accidental consumption and cleaning of the home were below (∼10^–7^) the benchmark limit, the annual risk of infection posed by *E. coli* for these two exposure scenarios exceeded the recommended benchmark limit (∼10^–3^). Additionally, the annual risk of infection posed by the intentional drinking of RHRW, was exceeded for both *E. coli* and *L. monocytogenes* (Figure 3A). The annual risk of infection for *M. tuberculosis* and adenovirus was only determined for two exposure scenarios: garden hosing (aerosol inhalation) and washing laundry by hand (aerosol ingestion volume was used as a substitute for aerosol inhalation volume to represent a worst-case scenario). Analysis of the mean annual risk of infection posed by *M. tuberculosis* in the RHRW samples indicated that both garden hosing (∼10^–5^) and washing laundry by hand (∼10^–5^) were below the annual infection risk benchmark limit (Figure 3B). In contrast, analysis of the mean annual risk of infection posed by adenovirus in the RHRW samples indicated that both garden hosing (∼10^–3^) and washing laundry by hand (∼10^–3^) exceeded the annual infection risk benchmark limit (Figure 3B).

## Discussion

Ethidium monoazide bromide-qPCR analyses indicated that intact (viable) cells of the human pathogens *L. monocytogenes* and *M. tuberculosis* were detected in all (100%) the RHRW samples, while *Yersinia* spp. were detected in 92% of the samples. *L. monocytogenes* has frequently been detected in soil, plant and surface water samples, as well as in sewage, slaughterhouse waste, human and animal faeces (Weis and Seeliger, 1975; Farber and Peterkin, 1991). Correspondingly, in a study conducted by Lyautey et al. (2007), *L. monocytogenes* was isolated repeatedly from surface water over a 5-month sampling period using a culture-based selective enrichment and isolation procedure. Similarly, Colburn et al. (1990) isolated *L. monocytogenes* from 62% (*n* = 37) of samples extracted from fresh or low-salinity river water, which drained into the Humboldt-Arcata Bay (California). These results emphasise the ability of *L. monocytogenes* to survive in environments that are characterised by physical (e.g., solar irradiation, temperature) and chemical (e.g., pH, oxygen concentration, nutrients) variation. Additionally, Linke et al. (2014) observed a significant link between the presence of *Listeria* spp. in soil samples (collected from 12 different areas in Austria from 2007 to 2009) and the abiotic conditions of the soil (e.g., pH, moisture, type of soil) and found that *Listeria* spp. were more regularly isolated from soil samples characterised by neutral pH, low moisture, or having a consistency made up of sand and humus. Moreover, the same authors noticed that seasonal changes had an effect on the prevalence of *Listeria* spp. in soil, with the lowest cell counts recorded in July. Several strains of *L. monocytogenes* have also been found to survive for months to several years in food processing plants, including those used to produce dairy, meat, fish and ready-to-eat products (Ferreira et al., 2014). It is thus hypothesised that the high frequency of detection of the ubiquitous organism *L. monocytogenes* in the rainwater samples, may be due to its ability to survive across a wide range of temperatures and resist several environmental stresses (Lyautey et al., 2007).

Similar to *L. monocytogenes*, *Yersinia* spp. are extensively distributed in the environment with common reservoirs identified as wild rodents, livestock, wild animals, water and soil (Kapperud, 1975; Mollaret et al., 1979). In 2014 and 2019 two outbreaks of yersiniosis were caused by *Y. enterocolitica* O9 (Norway) and *Y. enterocolitica* O3 (Sweden and Denmark), respectively, with both outbreaks linked to the consumption of fresh salad/vegetables (MacDonald et al., 2016; Espenhain et al., 2019). Traceback investigations into the outbreak linked to *Y. enterocolitica* O9 indicated that the factory did not regularly change the water in the rinsing tanks, used for the processing of the salad mixes, which was subsequently identified as the likely cause of the *Y. enterocolitica* O9 contamination of the food products (MacDonald et al., 2016). Interestingly, *Yersinia* spp. are carried by most mammals, but generally do not cause serological or histopathological responses in these hosts (Pocock et al., 2001). However, close contact between rodents (e.g., house mice) and humans or livestock, has resulted in rodents being identified as significant vectors of *Yersinia* spp. infection (Pocock et al., 2001). Therefore, the detection of *Yersinia* spp. in the RHRW samples is hypothesised to have originated from mice and other rodents’ faecal matter or bodily fluids being deposited on the catchment surface or in the rainwater harvesting tanks.

In comparison, while *M. tuberculosis* is capable of adapting to hostile or extreme environmental conditions (Cook et al., 2009), the presence and persistence of this bacterium in the environment, and its possible role in the cause and distribution of community-acquired tuberculosis, has been a continuous debate since the start of the 20th century (Velayati et al., 2015). However, it is known that transmission of this bacterium from the environment is possibly due to its ability to persist under various environmental conditions. For example, tuberculosis bacilli have been isolated from wooden tongue depressors over an 88-day time-period, woollen household carpet over a 19-day sampling period, and both dry and moist soil for up to 4 weeks post initial contamination (Velayati et al., 2015). Additionally, it has been suggested that water and soil can become contaminated with *M. tuberculosis* through sputum from infected individuals (coughing sputum) (Velayati et al., 2015). The presence of *M. tuberculosis* within the rainwater tank samples may thus have resulted from sputum contaminated soil, or other debris, being deposited into the rainwater harvesting tanks. This is a cause for serious concern as South Africa forms part of the top six countries around the world that are burdened by a high incidence of tuberculosis (Tadokera et al., 2020). Within the Overberg District Municipality (region where the sampling site is located), from 2011 to 2015, tuberculosis-related deaths were reported as the number one cause of mortality for individuals between 25 and 64 years of age (Health Systems Trust, 2019). Additionally, individuals living in poverty-stricken areas have been identified as being at higher risk of contracting tuberculosis, as these individuals generally live in crowded conditions and lack access to basic healthcare (Foster et al., 2015).

*Escherichia coli* and *Enterococcus* spp. are generally employed as indicators of faecal pollution by warm-blooded animals (Field and Samadpour, 2007; Harwood et al., 2014) and based on the high frequency of detection (100 and 99%, respectively) of these indicator organisms in the RHRW samples, the hypothesis that the rainwater may be contaminated with the faecal matter of rodents and animals, amongst others, that was deposited on the rooftops or in the gutter systems, is thus confirmed. However, as previously indicated, MST markers are frequently used in combination with traditional indicator organisms, as these markers are able to differentiate between several sources of faecal contamination. Amongst the most promising MST markers are members of the *Bacteroides* spp. as these organisms are limited to the digestive tract of both humans and warm-blooded animals, where they dominate in the natural gut microflora (Ravaliya et al., 2014) and are subsequently detected in high concentrations in host faecal matter (Fogarty et al., 2003; Ge et al., 2010). Of particular interest is the HF183 marker, which is conserved among *Bacteroides* strains of human origin and has exhibited high specificity for the detection of human faecal matter and sewage contamination in environmental waters (Harwood et al., 2014). A high frequency of the *Bacteroides* HF183 marker (76%, *n* = 25) was subsequently detected within the RHRW samples in the current study. Personal communication with a few residents of the Kleinmond Housing Scheme site indicated that in order to prevent pets from scavenging household waste, garbage bags are regularly placed on top of the rainwater harvesting tanks (Waso et al., 2016). It is thus hypothesised that household waste stored on top of the tanks could potentially have introduced human faecal matter (e.g., from babies nappies/diapers) into the rainwater tanks. Similarly, adenovirus and Lachnospiraceae are ubiquitously distributed in the environment, particularly in areas contaminated with sewage or human faeces (World Health Organisation, 2005; Newton et al., 2011). Therefore, the detection of adenovirus and Lachnospiraceae in the RHRW samples (96 and 80%, respectively) may have also occurred through the introduction of household waste into the rainwater tanks.

The MST markers (*Bacteroides* HF183, adenovirus and Lachnospiraceae) and traditional indicator organisms (*E. coli* and *Enterococcus* spp.) were then statistically correlated to the human pathogenic species (*M. tuberculosis*, *Yersinia* spp. and *L. monocytogenes*) detected in the rainwater. Results showed significant positive correlations for *E. coli* versus *L. monocytogenes* (*r* = 0.6738; *p* = 0.000); and *Enterococcus* spp. versus the *Bacteroides* HF183 marker (*r* = 0.4071; *p* = 0.043), while a significant negative correlation was observed for *M. tuberculosis* versus the *Bacteroides* HF183 marker (*r* = −0.4558; *p* = 0.022) (Table 3). The significant positive correlation recorded between *E. coli* and *L. monocytogenes*, could be explained by the fact that these organisms share several common reservoirs (e.g., water, soil, human and animal faeces) and could thus have entered the rainwater tank via a common source. Based on the cluster analysis (Figure 2), adenovirus was then also related to *L. monocytogenes* and *E. coli* (albeit not significantly), which is hypothesised to be due to the common occurrence of all three groups in faecal matter (Weis and Seeliger, 1975; Farber and Peterkin, 1991; Pocock et al., 2001; Waso et al., 2018). Similarly, the significant correlation and clustering observed between *Enterococcus* spp. and the *Bacteroides* HF183 marker confirms results of previous studies where indicator organisms positively correlated with MST markers (Korajkic et al., 2018; Waso et al., 2018). This is hypothesised to be due to the common occurrence of these indicators and MST markers in the gut of humans and warm-blooded animals, and consequently, in host faecal matter (Field and Samadpour, 2007; Ahmed et al., 2008a; Harwood et al., 2014). Interestingly, a significant negative correlation was observed between *M. tuberculosis* and the *Bacteroides* HF183 marker (*r* = −0.4558; *p* = 0.022). Research has indicated that during tuberculosis infection and the implementation of subsequent treatment strategies, the gut microbiota is altered significantly (Namasivayam et al., 2018). Consequently, a decrease in the diversity of *Bacteroides* spp. present in the gut has been observed during *M. tuberculosis* infection (Namasivayam et al., 2018), which could possibly elucidate the significant negative correlation observed between *M. tuberculosis* and the *Bacteroides* HF183 marker in the current study.

A QMRA framework was then applied to assess the health risk associated with the consumption of RHRW containing pathogenic *E. coli*, adenovirus, *L. monocytogenes* and *M. tuberculosis* for potable and several domestic activities (exception of *M. tuberculosis* and adenovirus where only two potential inhalation exposure scenarios were assessed). Results of the QMRA for *L. monocytogenes* indicated that the annual benchmark for infection risk was only exceeded for intentional drinking, while the risk associated with the use of the rainwater contaminated with *L. monocytogenes* for each of the remaining domestic activities was below the annual infection risk benchmark limit (<1 × 10^–4^). Similarly, for *E. coli*, the risk associated with the use of the RHRW for the domestic activities garden hosing, garden work, washing laundry by hand and washing/bathing, was below the annual infection risk benchmark limit. In contrast, the risk associated with intentional drinking, accidental consumption and cleaning of the home exceeded the annual infection risk benchmark limit (1 × 10^–4^) for untreated rainwater and thus posed a possible risk of infection by *E. coli*. This is concerning as results of a social survey conducted by Dobrowksy et al. (2014) indicated that 70% of individuals residing in the Kleinmond Housing Scheme site use the RHRW for cleaning, whilst 24% use it for drinking (without treatment). Consumption of rainwater contaminated with enteroinvasive *E. coli* pathotypes and *L. monocytogenes* could therefore significantly increase the occurrence of gastrointestinal disease in developing countries, particularly in sub-Saharan Africa (Ryan et al., 2014; Robins-Browne et al., 2016). Moreover, it should be noted that while the *L. monocytogenes* sequence type 6 subtype (predominantly associated with the major listeriosis outbreak in South Africa in 2017 and 2018) (Smith et al., 2019) was not analysed for in the current study, consumption of untreated RHRW could potentially lead to an increase in the number of listeriosis cases in the end-user community.

The annual risk of infection for adenovirus and *M. tuberculosis* was only calculated for two exposure scenarios (i.e., garden hosing and washing laundry by hand) which are linked to the inhalation of water particles, as infection with human adenovirus and *M. tuberculosis* bacilli primarily results in respiratory infections rather than gastrointestinal illness (World Health Organisation, 2005; Fennelly and Jones-López, 2015). Although it is possible to contract *M. tuberculosis* by consuming water contaminated with this bacterium, tuberculosis infection is initiated when droplet nuclei containing *M. tuberculosis* are inhaled and reach the alveoli of the lungs (Centers for Disease Control and Prevention, 2016). While the QMRA for *M. tuberculosis*, for garden hosing and washing laundry by hand, was below the annual infection risk benchmark limit, the QMRA for adenovirus exceeded the annual infection risk benchmark limit for both garden hosing (∼10^–3^) and washing laundry by hand (∼10^–3^). Adenovirus was selected for the QMRA analysis as this group of viruses are prevalent in high numbers in a wide range of water environments and have shown to be highly resistant to processes of disinfection and purification (Van Heerden et al., 2003). However, while the results obtained for *M. tuberculosis* are similar to data obtained by Hamilton et al. (2017), who observed that the annual risk of infection posed by the *Mycobacterium avium* complex (a group of bacteria related to *M. tuberculosis*), was below the benchmark value of 1 × 10^–4^, the health risks posed by *M. tuberculosis* in rainwater need to be further investigated. A significantly high incidence of tuberculosis is reported for the Western Cape region of South Africa and immune-compromised individuals have been identified as highly vulnerable to infection with *M. tuberculosis* (Sester et al., 2014). The *M. tuberculosis* QMRA results obtained in the current study may thus be an underestimation of the risk associated for immune-compromised individuals residing in the end-user communities, who rely on RHRW as a primary water source.

## Conclusion

The frequent detection of *L. monocytogenes*, *M. tuberculosis* and *Yersinia* spp. in the RHRW samples verifies that human pathogenic species are able to survive in rainwater which can pose a serious health risk to low- and middle-income communities, who routinely utilise RHRW as a sustainable water source. In addition, results of the correlation analysis confirm that traditional indicator organisms and MST markers should be used in combination to monitor RHRW quality, as both indicator groups correlated with the human pathogens (i.e., *E. coli* versus *L. monocytogenes*; *M. tuberculosis* versus *Bacteroides* HF183), as well as with each other (i.e., *Enterococcus* spp. versus *Bacteroides* HF183). Nonetheless, additional research should be conducted to assess the correlation of a broader range of human pathogenic species to the presence of several indicator organism groups (e.g., total coliforms, faecal coliforms) and MST markers (e.g., polyomavirus, *Bifidobacterium* spp., human mitochondrial DNA), in order to fully elucidate the environmental distribution and relationships between the various indicator groups and human pathogens.

The QMRA analysis then indicated that the use of RHRW containing *L. monocytogenes*, adenovirus, and *E. coli* poses a health risk to end-user communities, particularly when used for intentional drinking (*E. coli* and *L. monocytogenes*), cleaning of the home (*E. coli*), garden hosing (adenovirus), washing laundry by hand (adenovirus), or when accidentally consumed (*E. coli*). However, while the QMRA results indicated that the concentration of *M. tuberculosis* obtained in the current study did not pose a health-risk to the end-user community, further research should be conducted, taking into consideration the approximate percentage of immune-compromised individuals living in South Africa and who utilise RHRW, in order to accurately estimate the risk associated with the use of RHRW for potable and domestic activities. This can ultimately determine or predict the potential of various available point-of-use treatment technologies (e.g., filtration, solar disinfection, chlorination, solar pasteurisation) to effectively reduce the estimated health risk to within the benchmark limit.

## Data Availability Statement

The raw data supporting the conclusions of this article will be made available by the authors, without undue reservation.

## Author Contributions

JD, BR, and WK conceived and designed the experiments. JD performed the experiments. JD, BR, and MW analysed the data. WK and SK contributed reagents, materials, and analysis tools. JD, BR, MW, and WK compiled the manuscript. SK edited the manuscript. All authors contributed to the article and approved the submitted version.

## Conflict of Interest

The authors declare that the research was conducted in the absence of any commercial or financial relationships that could be construed as a potential conflict of interest.
