# Supplementary material for: Human Pathogenic Bacteria Detected in Rainwater: Risk Assessment and Correlation to Microbial Source Tracking Markers and Traditional Indicators
Source: Front Microbiol. 2021 May 7;12:659784. doi: 10.3389/fmicb.2021.659784 (PMC8138566; doi:10.3389/fmicb.2021.659784)
Supplement: Supplementary file 1 [file Presentation_1.zip › Supplementary material.docx]

**Supplementary material for the article: “Human Pathogenic Bacteria Detected in Rainwater: Risk Assessment and Correlation to Microbial Source Tracking Markers and Traditional Indicators”**

**Table S1** Physico-chemical parameters of the RHRW samples collected from different houses over the five-week sampling period (range per RHRW tank with the average included in brackets).

| **House** | **Temperature (˚C)** | **pH** | **Total dissolved solids (ppt)** | **Electrical conductivity (mS/m)** | **Dissolved oxygen (mg/L)** |
| --- | --- | --- | --- | --- | --- |
| A | 9.0 to 14.0  (11.8 ± 1.8) | 6.55 to 6.76  (6.68 ± 0.08) | 0.05 to 0.07  (0.06 ± 0.01) | 0.11 to 0.14  (0.13 ± 0.01) | 7.00 to 9.31  (8.11 ± 1.05) |
| B | 10.0 to 19.0  (13.9 ± 3.3) | 6.63 to 6.79  (6.71 ± 0.06) | 0.05 to 0.07  (0.06 ± 0.01) | 0.10 to 0.14  (0.12 ± 0.02) | 6.70 to 8.32  (7.38 ± 0.64) |
| C | 10.0 to 16.0  (12.9 ± 2.1) | 6.64 to 6.77  (6.70 ± 0.05) | 0.04 to 0.06  (0.05 ± 0.01) | 0.09 to 0.13  (0.11 ± 0.02) | 7.00 to 9.06  (7.90 ± 1.01) |
| D | 9.0 to 15.5  (12.3 ± 2.4) | 6.72 to 6.83  (6.77 ± 0.05) | 0.05 to 0.09  (0.07 ± 0.02) | 0.10 to 0.14  (0.12 ± 0.02) | 7.10 to 9.34  (7.77 ± 0.91) |
| E | 10.0 to 18.0  (12.5 ± 2.7) | 6.74 to 6.95  (6.83 ± 0.08) | 0.05 to 0.07  (0.06 ± 0.01) | 0.10 to 0.13  (0.12 ± 0.01) | 7.00 to 8.99  (7.70 ± 0.78) |

**Figure S1** Daily rainfall and ambient temperature recorded over the five-week sampling period (12 August 2020 to 9 September 2020). Sampling events are indicated by numbered red circles.

**Identity Confirmation of Positive Controls**

*E. coli* ATCC 13706, *Enterococcus faecalis* (clinical isolate), *L. monocytogenes* ATCC 13932 and *Yersinia enterocolitica* subsp. *enterocolitica* ATCC 27729 were inoculated into 5 mL Luria Bertani (LB) broth and incubated on a rotary wheel at 37 ºC for 12 to 18 hours. Two mL of each culture was then centrifuged at 16 000 × g for 10 min and the resulting pellet used for genomic DNA extractions. For the organisms/MST markers which positive control stains were not available for, DNA was extracted from wastewater. Briefly, 4 mL of influent wastewater collected from the Stellenbosch Wastewater Treatment Plant was centrifuged at 16 000 × g for 10 min and the resulting pellet used for genomic DNA extractions.Genomic DNA was extracted from the pellets using the *Quick*-DNA™ Faecal/Soil Microbe Miniprep Kit (Zymo Research, Irvine, USA) as per the manufacturer’s instructions.

The extracted DNA from the ATCC strains, *E. faecalis* (clinical isolate) and the positive controls from wastewater (*M. tuberculosis,* Lachnospiraceae and *Bacteroides* HF183) were subsequently used in species-specific conventional PCR as a template using the primer sets and PCR cycling parameters as outlined in **Table 1 (main manuscript file)**. The PCR reaction mixture (total volume of 25 µL) consisted of Green GoTaq^®^ Flexi Buffer (1X) (Promega), 2.0 mM MgCl_2_ (except for *E. coli* and *Enterococcus* spp. where 1 mM was used) (Promega), 0.2 mM of each dNTP (except for *Yersinia* spp. where 1 mM was used) (Thermo Scientific), the concentrations of the respective PCR primers as reported in **Table 1 (main manuscript file)**, 1.5 U of GoTaq^®^ Flexi DNA Polymerase [except for *Bacteroides* HF183 (2.5 U), *Enterococcus* spp. (1.25 U), *E. coli* (1.25 U) and adenovirus (1.25 U)] (Promega), and 5 μL of template DNA. Following amplification, the PCR products were visualised using gel electrophoresis [80 Volts (V) for 80 minutes] on a 1% (w/v) agarose (SeaKem^®^ LE Agarose, Lonza) gel, in a 1X tris/acetate/ethylenediaminetetraacetic acid (TAE) buffer containing 0.5 µg/mL ethidium bromide (EtBr). The amplicon size for each isolate was then determined using the Generuler™ 1 kb Plus DNA ladder (ThermoFischer Scientific). Following visualisation, each of the amplicons were purified and concentrated using the Wizard® SV Gel and PCR Clean-up System (Promega Corp), as per the manufacturer’s instructions. The purified, concentrated PCR products were sent for sequencing at the Central Analytical Facilities (CAF) at Stellenbosch University. The FinchTV version 1.4.0 software was used to examine the chromatogram of each sequence, whereafter sequence identification for each isolate was carried out using the National Centre for Biotechnology Information (NCBI), Basic Local Alignment Search Tool (BLAST), available at <https://blast.ncbi.nlm.nih.gov/Blast.cgi> (Altschul et al., 1990). The adenovirus positive control was a lyophilised adenovirus sample obtained from Coris Bioconcept (Gembloux, Belgium) and therefore was not sequenced.

.

**Table S2** Sequencing results to confirm the identity of the positive controls used in the current study.

| **Target** | **BLAST result** | **Sequence Similarity (%)** | **Accession Number** |
| --- | --- | --- | --- |
| *Yersinia* spp. | *Yersinia enterocolitica* subsp. *enterocolitica* strain NCTC12982 genome assembly, chromosome 1 | 100 | LR5904769.1 |
| *M. tuberculosis* | *Mycobacterium tuberculosis* strain FDAARGOS_756 chromosome, complete genome | 99.55 | CP054014.1 |
| *L. monocytogenes* | *Listeria monocytogenes* strain PNUSAL000009 chromosome | 100 | CP054042.1 |
| *E. coli* | *Escherichia coli* strain EA13 chromosome, complete genome | 95.24 | CP069712.1 |
| *Enterococcus* spp. | *Enterococcus faecalis* strain 133170041-3 chromosome, complete genome | 100 | CP046108.1 |
| *Lachnospiraceae* | Uncultured *Lachnospiraceae* bacterium clone DogPlate_1_H12 16S ribosomal RNA gene, partial sequence | 100 | MG702965.1 |
| *Bacteroides* HF183 | *Bacteroides doreii* partial 16S rRNA gene strain VPI-3776A | 100 | LR999587.1 |

**Table S3** Exposure scenarios for the use of RHRW under investigation in the current study as outlined by Reyneke et al. (2020).

|  | **Activity** | **Volume (distribution)** | **Frequency/year**  **(distribution)** | **Reference** |
| --- | --- | --- | --- | --- |
| 1 | Washing laundry by hand  (Aerosol ingestion) | A_v_: 1.13 x 10^-13^/mL to 8.18 x 10^-12^/mL (Uniform)  A_L_: 0 to 1.07 x 10^5^/L (Uniform)  T: 60 min (Point)  B_R:_ 10 L/min to 20 L/min (Uniform) | 35 to 55 (Uniform) | Fischer et al. (2019) |
| 2 | Cleaning of the home  (Hand to mouth contact) | F_T_: µ = 20 µm, σ = 10 µm (Normal)  A_HS_: 0.106 m^2^ to 0.131 m^2^ (Uniform)  S_H_: 0.1 (Point)  TE_HF_: 0.68 (*E. coli*, Point), 0.34 to 0.68 (Other, Uniform)  C_E_: 5 to 20 (Uniform) | 26 to 104 (Uniform) | USEPA (2011)  Julian and Pickering (2015)  Rusin et al. (2002) |
| 3 | Garden hosing  (Aerosol ingestion)  (Aerosol inhalation) | 0.002 µL to 1.1 mL (Uniform)  0.008 to 0.5 µL (Uniform) | 90 to 180 (Uniform) | Ahmed et al. (2010) |
| 4 | Garden work  (Hand to mouth contact) | 0.5 to 2 mL (Uniform) | 4 to 35 (Uniform) | Busgang et al. (2018) |
| 5 | Washing/bathing  (Hand to mouth contact) | F_T_: µ = 1.65 µm, σ = 0.32 µm (Normal)  A_HS_: 0.106 m^2^ to 0.131 m^2^ (Uniform)  S_H_: 0.1 (Point)  TE_HF_: 0.68 (*E. coli*, Point), 0.34 to 0.68 (Other, Uniform)  C_E_: 5 to 20 (Uniform) | 365 (Point) | USEPA (2011)  Julian and Pickering (2015)  Rusin et al. (2002) |
| 6 | Intentional drinking | 872.5 mL (Poisson) | 365 (Point) | Mons et al. (2007) |
| 7 | Accidental consumption | 50 to 200 mL (Uniform) | 0.5 to 2 (Uniform) | Busgang et al. (2018) |

F_T_: water film thickness; S_H_: surface area of hand in contact with mouth; A_HS_: average surface area of hand; C_E_: hand-to-mouth contact events; TE_HF_: transfer efficiency of bacteria; A_v_: aerosol volume; B_R_: human breathing rate; A_L_: aerosol concentration; T: time.

**Table S4** Performance characteristics of the respective qPCR assays utilised to quantify *Bacteroides* HF183, adenovirus, Lachnospiraceae, *E.*  *coli*, *Enterococcus* spp., *L. monocytogenes*, *M. tuberculosis* and *Yersinia* spp.

| **qPCR Assay** | **Amplification efficiency (*E*)** | **Slope** | **y-intercept** | **Correlation coefficient (*r*^2^)** |
| --- | --- | --- | --- | --- |
| *Bacteroides* HF183 | 1.91 (96%) | -3.5682 | 36.41 | 1.00 |
| Adenovirus | 2.20 (110%) | -2.9194 | 31.29 | 0.98 |
| Lachnospiraceae | 1.60 (80%) | -4.9253 | 50.28 | 1.00 |
| *E. coli* | 1.90 (95%) | -3.5973 | 36.03 | 1.00 |
| *Enterococcus* spp. | 1.98 (99%) | -3.3300 | 33.36 | 1.00 |
| *L. monocytogenes* | 1.92 (96%) | -3.5352 | 36.74 | 1.00 |
| *M. tuberculosis* | 1.92 (96%) | -3.5409 | 35.10 | 1.00 |
| *Yersinia* spp. | 1.92 (96%) | -3.5258 | 32.87 | 1.00 |

**References:**

Altschul, S.F., Gish, W., Miller, W., Myers, E.W., Lipman, D.J. (1990). Basic local alignment search tool. J. Mol. Biol. 215:3, 403-410. <https://blast.ncbi.nlm.nih.gov/Blast.cgi>.

Ahmed, W., Vieritz, A., Goonetilleke, A., Gardner, T. (2010). Health risk from the use of roof harvested rainwater in Southeast Queensland, Australia, as potable or nonpotable water, determined using quantitative microbial risk assessment. Appl. Environ. Microbiol. 76, 7382-7391. doi.org/10.1128/AEM.00944-10.

Busgang, A., Friedler, E., Gilboa, Y., Gross, A. (2018). Quantitative microbial risk analysis for various bacterial exposure scenarios involving greywater reuse for irrigation. Water. 10:4, 413. doi.org/10.3390/w10040413.

Fischer, F., Hamdan, Y., Hansson, A., Josefsson, E., Sundström, L. (2019). Water quality modelling and quantitative microbial risk assessment of Msunduzi river. [dissertation/bachelor’s thesis]. [Gothenberg (SE)]: Chalmers University of Technology.

Julian, T.R., and Pickering, A.J. (2015). A pilot study on integrating videography and environmental microbial sampling to model fecal bacterial exposures in peri-urban Tanzania. PLoS ONE. 10, e0136158. doi.org/10.1371/journal.pone.0136158.

Mons, M.N., Van Der Wielen, J.M.L., Blokker, E.J.M., Sinclair, M.I., Hulshof, K.F.A.M., Dangendorf, F., et al. (2007). Estimation of the consumption of cold tap water for microbiological risk assessment: an overview of studies and statistical analysis of data. J. Water Health. 5, 151-170. doi.org/10.2166/wh.2007.141.

Reyneke, B.*,* Hamilton, K.A., Fernández-Ibáñez, P., Polo-López, M.I., McGuigan, K.G., Khan, S., et al. (2020). EMA-Illumina 16S metagenomic sequencing informs risk assessment analysis of water treatment systems. Sci. Total Environ. 743, 140717. doi.org/10.1016/j.scitotenv.2020.140717.

Rusin, P., Maxwell, S., Gerba, C. (2002). Comparative surface-to-hand and fingertip-to-mouth transfer efficiency of gram-positive bacteria, gram-negative bacteria, and phage. J. Appl. Microbiol. 93, 585-592. doi.org/10.1046/j.1365-2672.2002.01734.x.

United States Environmental Protection Agency (USEPA). (2011). Exposure Factors Handbook: 2011 Edition. https://cfpub.epa.gov/ncea/risk/recordisplay.cfm?deid=236252 [accessed October 29, 2020].
